# Characterization of the Exopolysaccharide Biosynthesis Pathway in Myxococcus xanthus

**DOI:** 10.1128/JB.00335-20

**Published:** 2020-09-08

**Authors:** María Pérez-Burgos, Inmaculada García-Romero, Jana Jung, Eugenia Schander, Miguel A. Valvano, Lotte Søgaard-Andersen

**Affiliations:** a Max Planck Institute for Terrestrial Microbiology, Department of Ecophysiology, Marburg, Germany; b Wellcome-Wolfson Institute for Experimental Medicine, Queen's University Belfast, Belfast, United Kingdom; Geisel School of Medicine at Dartmouth

**Keywords:** *Myxococcus xanthus*, Wzx flippase, Wzy polymerase, development, exopolysaccharide, fruiting body formation, motility, polysaccharide, sporulation, type IV pili

## Abstract

Myxococcus xanthus arranges into two morphologically distinct biofilms depending on its nutritional status, i.e., coordinately spreading colonies in the presence of nutrients and spore-filled fruiting bodies in the absence of nutrients. A secreted polysaccharide, referred to as exopolysaccharide (EPS), is a structural component of both biofilms and is also important for type IV pilus-dependent motility and fruiting body formation. Here, we characterize the biosynthetic machinery responsible for EPS biosynthesis using bioinformatics, genetics, heterologous expression, and biochemical experiments. We show that this machinery constitutes a Wzx/Wzy-dependent pathway dedicated to EPS biosynthesis. Our data support that EpsZ (MXAN_7415) is the polyisoprenyl-phosphate hexose-1-phosphate transferase responsible for the initiation of the repeat unit synthesis. Heterologous expression experiments support that EpsZ has galactose-1-P transferase activity. Moreover, MXAN_7416, renamed Wzx_EPS_, and MXAN_7442, renamed Wzy_EPS_, are the Wzx flippase and Wzy polymerase responsible for translocation and polymerization of the EPS repeat unit, respectively. In this pathway, EpsV (MXAN_7421) also is the polysaccharide copolymerase and EpsY (MXAN_7417) the outer membrane polysaccharide export (OPX) protein. Mutants with single in-frame deletions in the five corresponding genes had defects in type IV pilus-dependent motility and a conditional defect in fruiting body formation. Furthermore, all five mutants were deficient in type IV pilus formation, and genetic analyses suggest that EPS and/or the EPS biosynthetic machinery stimulates type IV pilus extension. Additionally, we identify a polysaccharide biosynthesis gene cluster, which together with an orphan gene encoding an OPX protein make up a complete Wzx/Wzy-dependent pathway for synthesis of an unknown polysaccharide.

**IMPORTANCE** The secreted polysaccharide referred to as exopolysaccharide (EPS) has important functions in the social life cycle of M. xanthus; however, little is known about how EPS is synthesized. Here, we characterized the EPS biosynthetic machinery and showed that it makes up a Wzx/Wzy-dependent pathway for polysaccharide biosynthesis. Mutants lacking a component of this pathway had reduced type IV pilus-dependent motility and a conditional defect in development. These analyses also suggest that EPS and/or the EPS biosynthetic machinery is important for type IV pilus formation.

## Abstract (precis)

The secreted polysaccharide referred to as exopolysaccharide (EPS) has important functions in the social life cycle of M. xanthus; however, little is known about how EPS is synthesized. Here, we characterized the EPS biosynthetic machinery and showed that it makes up a Wzx/Wzy-dependent pathway for polysaccharide biosynthesis. Mutants lacking a component of this pathway had reduced type IV pilus-dependent motility and a conditional defect in development. These analyses also suggest that EPS and/or the EPS biosynthetic machinery is important for type IV pilus formation.

## INTRODUCTION

Bacteria often exist in biofilms, which are surface-associated communities where cells are embedded in a self-produced extracellular matrix (1). Typically, this matrix is composed of proteins, extracellular DNA (eDNA), and polysaccharides (2). The polysaccharides serve several functions in a biofilm, including structural roles, hydration, adhesion to substrates, cohesion between cells, and protection against antibacterials, grazing, and bacteriophages (2–4).

The Gram-negative deltaproteobacterium Myxococcus xanthus is a model organism to study social behaviors in bacteria. Depending on their nutritional status, M. xanthus cells organize into two morphologically distinct biofilms (5, 6). In the presence of nutrients, cells grow, divide, and move across surfaces by means of two motility systems to generate colonies that are embedded in a polysaccharide referred to as exopolysaccharide (EPS) and in which cells at the colony edge spread outwards in a highly coordinated fashion (6–8). Under nutrient limitations, growth ceases and cells alter their motility behavior and begin to aggregate. The aggregation process culminates in the formation of mounds of cells inside which the rod-shaped cells differentiate into environmentally resistant spores, leading to the formation of mature fruiting bodies (5, 6). EPS also makes up a substantial part of individual fruiting bodies (9–11).

The two motility systems of M. xanthus are important for formation of both biofilms (12). One motility system depends on type IV pili (T4P), which are highly dynamic filaments that undergo cycles of extension, surface adhesion, and retraction. Retractions generate a force sufficient to pull a cell forward (13). The second system is for gliding motility and depends on the Agl/Glt complexes (6, 7). Generally, T4P-dependent motility involves the movement of groups of cells, while gliding motility involves the movement of individual cells (12, 14).

Besides its role as a structural component of spreading colonies and fruiting bodies, EPS in M. xanthus is also important for T4P-dependent motility (9, 15) and fruiting body formation (9, 10, 16–18). It has been proposed that EPS stimulates T4P-dependent motility by stimulating retraction of T4P (15, 19). Most insights into the function of EPS in M. xanthus have been obtained from analyses of regulatory mutants with altered levels of EPS synthesis. Among these mutants, the best studied include those of the Dif chemosensory system and the SgmT/DigR two-component system. The Dif system is a key regulator of EPS synthesis; analyses of *dif* (previously *dsp* [10, 20, 21]) mutants have shown that decreased EPS accumulation (18, 21, 22) causes defects in T4P-dependent motility and fruiting body formation (17, 18). While the phosphotransfer reactions within the Dif system have been described in detail (22, 23), it is unknown how the Dif system stimulates EPS synthesis. Similarly, mutants of the SgmT/DigR system in which DigR is a DNA-binding response regulator have increased EPS accumulation and reduced T4P-dependent motility, as well as a defect in fruiting body formation (24, 25). Transcriptome analyses support that the effect on EPS accumulation is not caused by direct effects on the expression of genes for EPS synthesis (25). Compared to the several identified regulators of EPS synthesis, relatively little is known about EPS biosynthesis. Here, we focused on the identification of proteins directly involved in EPS biosynthesis.

**FIG 1:**
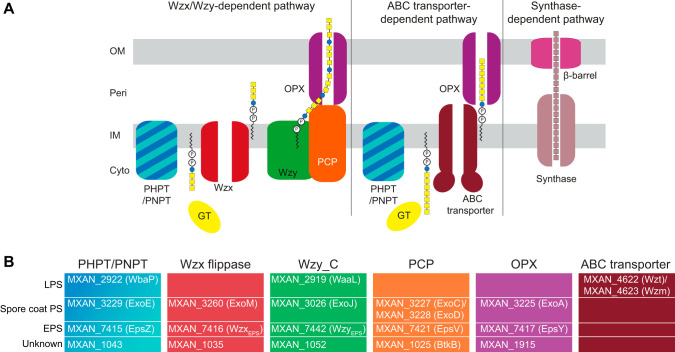
Identification of homologs of polysaccharide biosynthesis proteins in M. xanthus. (A) Schematic of the three pathways for polysaccharide biosynthesis in Gram-negative bacteria. (B) Bioinformatics-based identification of homologs of polysaccharide biosynthesis proteins in M. xanthus. Color code is the same as that used for panel A. Note that WaaL is the LPS O-antigen ligase (37), while the remaining three proteins with a Wzy_C domain are predicted polymerases.

Synthesis of bacterial cell surface polysaccharides can occur via three different pathways, the Wzx/Wzy-, ABC transporter-, or synthase-dependent pathway (26, 27) (Fig. 1A). In the Wzx/Wzy- and ABC transporter-dependent pathways, synthesis generally starts with the transfer of a sugar-1-P from a UDP-sugar to an undecaprenyl phosphate (Und-P) molecule in the inner leaflet of the inner membrane (IM) to form an Und-PP-sugar molecule (28). The priming enzymes are broadly classified in two groups, polyisoprenyl-phosphate hexose-1-phosphate transferases (PHPTs) and polyisoprenyl-phosphate *N*-acetylhexosamine-1-phosphate transferases (PNPTs) (29). Subsequently, the polysaccharide chain is elongated by the action of specific glycosyltransferases (GTs), and this depends on the specific pathway. In the Wzx/Wzy-dependent pathway, GTs synthesize the repeat unit of the polysaccharide on the cytoplasmic side of the IM; each unit is then translocated across the IM by the Wzx flippase and polymerized by the Wzy polymerase into a longer chain. Chain length is generally controlled by a Wzz protein, which belongs to the polysaccharide copolymerase (PCP) family and results in the formation of polysaccharide molecules with a range of lengths (30, 31). In contrast, in the ABC transporter-dependent pathway, the full-length polysaccharide chain is synthesized on the cytoplasmic side of the IM and is then translocated across the IM by an ABC transporter (32). In the synthase-dependent pathway, synthesis and translocation across the IM take place simultaneously by a multifunctional synthase protein complex in the IM (33). In the Wzx/Wzy- and ABC transporter-dependent pathways, the polysaccharide molecule reaches the cell surface by translocation through an outer membrane (OM) polysaccharide export (OPX) protein, and in the synthase-dependent pathway translocation occurs via an OM β-barrel protein (26, 33).

The *eps* locus in M. xanthus was identified by transposon mutagenesis and shown to encode homologs of proteins involved in polysaccharide biosynthesis (9). Moreover, several *eps* genes were identified as essential for EPS biosynthesis (9, 22, 34–36). Here, we searched the reannotated *eps* locus and the remaining M. xanthus genome for homologs of proteins for polysaccharide biosynthesis. We report that the *eps* locus encodes a complete Wzx/Wzy-dependent pathway for EPS biosynthesis. In-frame deletions in the corresponding genes specifically resulted in EPS biosynthesis defects, while these mutants still synthesized lipopolysaccharide (LPS) O antigen and spore coat polysaccharide and had a normal cell morphology. Phenotypic analysis of these mutants, including complementation experiments, demonstrated that they have a defect in T4P-dependent motility and conditional defects in development. In addition, we identify a polysaccharide biosynthesis gene cluster of unknown function that, together with an orphan gene encoding an OPX protein, encodes a complete Wzx/Wzy-dependent pathway for biosynthesis of a polysaccharide of unknown function.

## RESULTS

### Identification of homologs of proteins of Wzx/Wzy-dependent pathways for polysaccharide biosynthesis and export.

The M. xanthus genome encodes a total of 66 GTs (CAZy). Therefore, to identify genes for EPS biosynthesis, we searched the M. xanthus genome for homologs (see Materials and Methods) of the membrane components of the three biosynthesis pathways (Fig. 1A). We identified homologs encoding predicted proteins of the Wzx/Wzy and ABC-transporter pathways but none corresponding to a synthase-dependent pathway (Fig. 1B). Several of these homologs were previously shown to be important for LPS synthesis or spore coat polysaccharide biosynthesis (37–41) (Fig. 1B). Notably, none of these proteins is required for EPS biosynthesis. The MraY homolog (MXAN_5607), which belongs to the PNPT family and is involved in PG synthesis, was not considered here.

**FIG 2:**
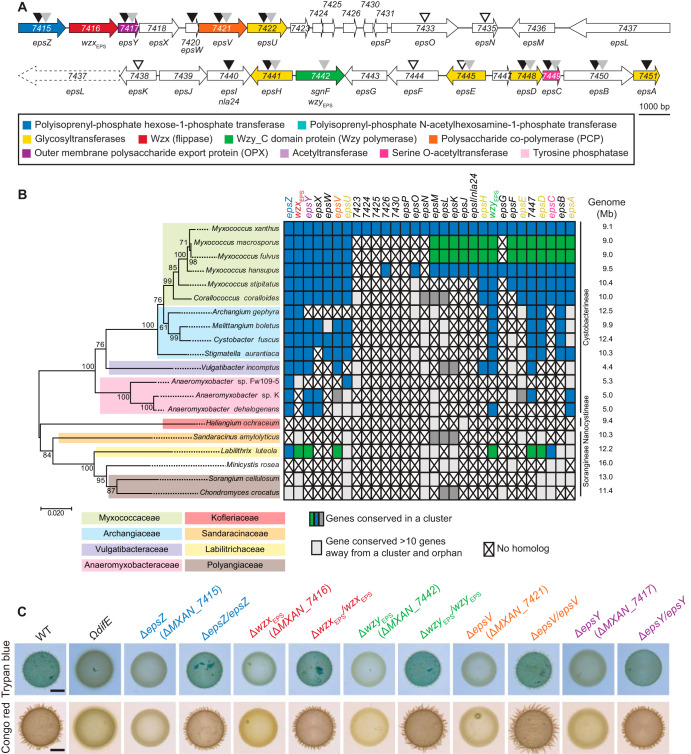
Bioinformatics and genetic analysis of the *eps* locus. (A) *eps* locus in M. xanthus. Genes are drawn to scale, and MXAN number or gene name is indicated (Table S1). The color code indicates predicted functions as indicated in the key and is used throughout. Black, gray, and white arrowheads indicate mutations previously reported to cause a defect in EPS synthesis (9, 22, 34, 36), a defect in T4P-dependent motility but with no test of EPS synthesis (42), and no effect on EPS synthesis (9), respectively. (B) Taxonomic distribution and synteny of *eps* genes in *Myxococcales* with fully sequenced genomes. A reciprocal best BLASTP hit method was used to identify orthologs. (Left) 16S rRNA tree of *Myxococcales* with fully sequenced genomes. (Right) Genome size and family and suborder classification are indicated. To evaluate gene proximity and cluster conservation, 10 genes was considered the maximum distance for a gene to be in a cluster. Genes found in the same cluster (within a distance of <10 genes) are marked with the same color (i.e., blue, green, and dark gray). Light gray indicates a conserved gene that is found somewhere else on the genome (>10 genes away from a cluster); a cross indicates no homolog found. (C) Determination of EPS synthesis. Twenty-microliter aliquots of cell suspensions of strains of the indicated genotypes at 7 × 10^9^ cells ml^−1^ were spotted on 0.5% agar supplemented with 0.5% CTT and Congo red or trypan blue and incubated for 24 h. In the complementation strains, the complementing gene was expressed ectopically from the native (*epsZ*, *wzx*_EPS_, and *wzy*_EPS_) or *pilA* promoter on a plasmid integrated in a single copy at the Mx8 *attB* site. The Ω*difE* mutant served as a negative control.

The reannotated *eps* locus consists of two gene clusters (*MXAN_7515-_7422* and *MXAN_7441-_7451*) that encode all the proteins of a complete Wzx/Wzy-dependent pathway (Fig. 2A; see also Table S1 in the supplemental material). Specifically, these two gene clusters encode homologs of a PHPT (EpsZ/MXAN_7415), a Wzx flippase (MXAN_7416), a Wzy polymerase (MXAN_7442, previously SgnF [42]), a PCP protein (EpsV/MXAN_7421), and an OPX protein (EpsY/MXAN_7417), as well as five GTs (EpsU/MXAN_7422, EpsH/MXAN_7441, EpsE/MXAN_7445, EpsD/MXAN_7448, and EpsA/MXAN_7451) and a serine *O*-acetyltransferase (EpsC/MXAN_7449). Previous genetic analyses using transposon insertions, plasmid insertions, or in-frame deletion mutants demonstrated that genes in both clusters are important for EPS synthesis (9, 22, 34, 35) (Fig. 2A). Genes in both clusters also were previously shown to be important for T4P-dependent motility without directly testing for EPS synthesis (42) (Fig. 2A). The two gene clusters are separated by 13 genes encoding proteins predicted not to be directly involved in polysaccharide synthesis (Fig. 2A; see Table S1 in the supplemental material). Consistent with this, genetic analyses for some of these genes confirmed that they are not important for EPS synthesis (9), except for *MXAN_7440* (Nla24/EpsI), which encodes a c-di-GMP binding NtrC-like transcriptional regulator (36, 43) that is phosphorylated by the histidine kinase MXAN_7439 (44).

In a bioinformatics approach searching for orthologs of the proteins encoded by the entire *eps* locus in all fully sequenced *Myxococcales* genomes and using a reciprocal best BLASTP hit method, as described previously (41), we found that the two gene clusters encoding proteins for polysaccharide synthesis (*MXAN_7415-MXAN_7422* and *MXAN_7442-MXAN_7451*) are largely conserved in closely related *Cystobacterineae* (Fig. 2B). Importantly, in several of these genomes, the two clusters are present in a single uninterrupted gene cluster (e.g., *M. stipitatus* and Stigmatella aurantiaca) (Fig. 2B). Interestingly, in *M. macrosporus* and *M. fulvus*, the two gene clusters are separated by a set of genes that are conserved between these two organisms but not homologous to the genes separating the two clusters in M. xanthus. Together, based on previous genetic analyses and because genes for polysaccharide biosynthesis are often clustered (45), our data support that the two separated gene clusters in the M. xanthus *eps* locus encode a Wzx/Wzy-dependent pathway for EPS synthesis.

**FIG 3:**
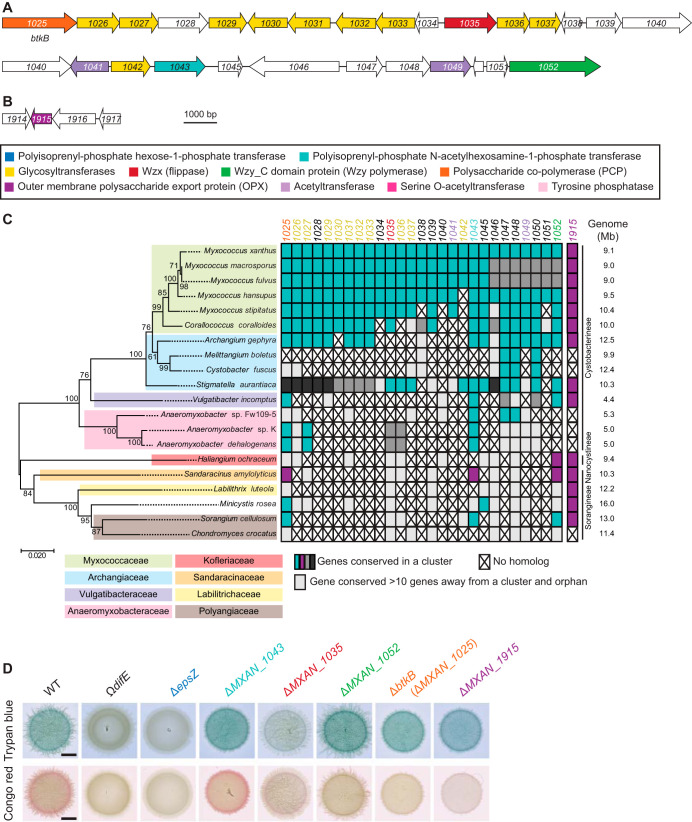
Bioinformatics and genetic analysis of the *MXAN_1025-1052/_1915* loci. (A and B) *MXAN_1025-1052* and *_1915* loci in M. xanthus. Genes are drawn to scale, and the MXAN number or gene name is indicated (Table S2). The color code indicates predicted functions as indicated in the key and is used throughout. (C) Taxonomic distribution and synteny of genes in the *MXAN_1025-1052/_1915* loci in *Myxococcales* with fully sequenced genomes. A reciprocal best BLASTP hit method was used to identify orthologs. (Left) 16S rRNA tree of *Myxococcales* with fully sequenced genomes. (Right) Genome size, family, and suborder classification are indicated. To evaluate gene proximity and cluster conservation, 10 genes was considered the maximum distance for a gene to be in a cluster. Genes found in the same cluster (within a distance of <10 genes) are marked with the same color (i.e., cyan, magenta, and dark and medium gray). Light gray indicates a conserved gene that is found somewhere else on the genome (>10 genes away from a cluster); a cross indicates no homolog found. (C) Determination of EPS synthesis. Twenty-microliter aliquots of cell suspensions of strains of the indicated genotypes at 7 × 10^9^ cells ml^−1^ were spotted on 0.5% agar supplemented with 0.5% CTT and Congo red or trypan blue and incubated 24 h. The Ω*difE* mutant served as a negative control. Scale bars, 3 μm.

We also identified a second locus encoding homologs of a Wzx/Wzy pathway (Fig. 3A and Table S2). Specifically, this locus encodes homologs of a PNPT (MXAN_1043), a Wzx flippase (MXAN_1035), a Wzy polymerase (MXAN_1052), a Wzc chain length regulator (MXAN_1025 or BtkB [46]) of the PCP-2, 10 GTs (MXAN_1026, MXAN_1027, MXAN_1029, MXAN_1030, MXAN_1031, MXAN_1032, MXAN_1033, MXAN_1036, MXAN_1037, and MXAN_1042), and two acetyltransferases (MXAN_1041 and MXAN_1049). Finally, we identified a gene encoding an OPX protein (MXAN_1915) that is not part of a gene cluster encoding proteins involved in polysaccharide synthesis (Fig. 3B and Table S2). Using bioinformatics, as described above, we found that the large gene cluster as well as *MXAN_1915* are conserved in closely related *Cystobacterineae* (Fig. 3C). Importantly, the *MXAN_1915* ortholog of *Sandaracinus amylolyticus* is found in a cluster with homologs of *MXAN_1025*, *MXAN_1043*, and *MXAN_1052*. Because the *MXAN_1025-_1052* locus does not encode an OPX homolog, these observations support that MXAN_1915 could function together with the proteins encoded by this locus, and together they would make up a complete Wzx/Wzy-dependent pathway for biosynthesis of a polysaccharide. Based on these analyses, we hypothesized that the proteins encoded by the *eps* locus and the proteins encoded by the *MXAN_1025-_1052*/*_1915* loci make up two independent and dedicated pathways for polysaccharide synthesis.

### The *eps* locus is essential for EPS biosynthesis.

To test for the importance of genes of the *eps* locus and the *MXAN_1025-_1052/_1915* loci for EPS synthesis, we generated 10 in-frame deletions in genes encoding the five conserved core components of Wzx/Wzy-dependent pathways (i.e., the genes for the PH/NPT, Wzx, Wzy, PCP, and OPX). Subsequently, we used plate-based colorimetric assays with either Congo red or trypan blue to assess EPS biosynthesis. As a negative control, we used a Ω*difE* mutant, which has a defect in EPS synthesis (17).

All five mutations in the *eps* locus abolished EPS synthesis (Fig. 2C). Importantly, the EPS synthesis defects of these five Δ*eps* mutants were complemented by ectopic expression of the relevant full-length gene from a plasmid integrated in a single copy at the Mx8 *attB* site (Fig. 2C). In contrast, in the case of the five in-frame deletions in the genes of the *MXAN_1025-_1052*/*_1915* loci, only the Δ*MXAN_1035* mutant, which lacks a Wzx flippase homolog (Fig. 3A to C), caused a significant decrease in EPS synthesis. Based on several arguments, we do not think that MXAN_1035 is directly involved in EPS synthesis but rather that the Δ*MXAN_1035* mutation results in titration of Und-P.

First, mutation of *MXAN_7416*, which codes for a Wzx flippase homolog in the *eps* locus, completely blocked EPS synthesis (Fig. 2), supporting that MXAN_7416 is the flippase involved in EPS biosynthesis and that MXAN_1035 cannot replace MXAN_7416 flippase function. Second, as mentioned, enzymes of the same polysaccharide biosynthesis and export pathway are typically encoded in the same locus (45); however, the three other mutations in the *MXAN_1025-_1052* locus did not have a significant effect on EPS biosynthesis (Fig. 3D). Third, blocking translocation of a specific sugar unit across the IM can cause sequestration of Und-P and thereby result in pleiotropic effects on the synthesis of other polysaccharides (47–50). Consistent with this, a Δ*MXAN_1035* mutation was previously shown to cause a reduction in glycerol-induced sporulation (see below), likely by interfering with spore coat polysaccharide biosynthesis (39); however, MXAN_3260 (ExoM) was recently shown to be the flippase involved in spore coat polysaccharide synthesis (41). Although we cannot completely rule out that MXAN_1035 is involved in EPS synthesis, these considerations support that it is unlikely that MXAN_1035 is part of the EPS biosynthesis machinery. In total, our results suggest that the *eps* locus encodes homologs of a Wzx/Wzy-dependent pathway for EPS biosynthesis. Therefore, we renamed MXAN_7416 and MXAN_7442 to Wzx_EPS_ and Wzy_EPS_. From here on, we focused on the five core components of the Wzx/Wzy-dependent pathway for EPS synthesis.

### Δ*eps* mutants synthesize spore coat polysaccharide and LPS and have normal cell morphology.

In addition to EPS, M. xanthus synthetizes O-antigen LPS (51) and a spore coat polysaccharide (52). As mentioned, because blocking the synthesis of one polysaccharide can affect the synthesis of other polysaccharides, including PG, by sequestration of Und-P through accumulation of Und-PP intermediates, we determined whether the lack of the EPS biosynthetic proteins affects spore coat polysaccharide, LPS, or PG synthesis.

**FIG 4:**
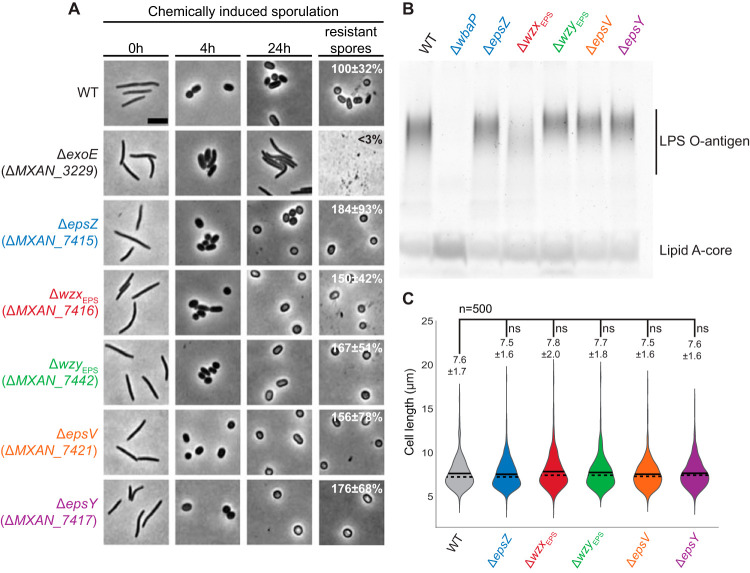
Phenotypic characterization of the Δ*eps* mutants. (A) Chemically induced sporulation. Sporulation was induced by addition of glycerol to a final concentration of 0.5 M. At 0, 4, and 24 h after glycerol addition, cell morphology was documented. In images labeled resistant spores, cells were exposed to sonic and heat treatment before microscopy. Sporulation frequency after sonic and heat treatment is indicated as the mean from three biological replicates relative to the WT ± standard deviations. Scale bar, 5 μm. (B) Extracted LPS from the same number of cells was separated by SDS-PAGE and detected with Pro-Q Emerald 300. (C) Cell length measurements of the Δ*eps* mutants. Cell length is shown in a violin plot, which indicates the probability density of the data at different cell length values. *n *= 500 combined from two biological replicates, and mean and median values are represented by a continuous and dashed line, respectively. Samples were compared using a Mann-Whitney test; ns, not significant.

Synthesis of the spore coat polysaccharide is essential for sporulation in M. xanthus (40, 53). To evaluate whether the Δ*eps* mutants synthetized spore coat polysaccharide, we analyzed sporulation independently of starvation. For this, we profited from an assay in which sporulation occurs rapidly and synchronously and is induced chemically by the addition of glycerol at a high concentration (0.5 M) to cells growing in nutrient-rich broth (54). In response to adding glycerol, cells of wild type (WT) and all five *eps* in-frame deletion mutants rounded up during the first 4 h and had turned into phase-bright resistant spores by 24 h (Fig. 4A). Cells of the Δ*exoE* mutant, which lacks the PHPT for initiating spore coat polysaccharide biosynthesis, were used as a negative control (39, 41), remained rod-shaped, and did not form phase-bright spores. Interestingly, the sporulation efficiency of all five Δ*eps* mutants was increased compared to that of the WT (Fig. 4A). Because the spores formed by the WT under high concentrations of glycerol adhere to glass surfaces and each other, forming large aggregates, while the spores formed by the Δ*eps* mutants do not, we speculate that the ease of harvesting the EPS̄ spores rather than the *eps* mutations *per se* results in an apparent increase in the overall sporulation efficiency. We conclude that lack of the EPS biosynthetic proteins does not cause a sporulation defect, in agreement with previous observations that mutation of *epsV* did not affect glycerol-induced sporulation (39).

LPS in total cell extracts was detected by Emerald staining and the Δ*wbaP* mutant, which lacks the PHPT for O-antigen biosynthesis, served as a negative control (37). A fast-running lipid-A core band and polymeric LPS O-antigen bands were detected in LPS preparations of WT and the five Δ*eps* mutants, while only the lipid A core band was detected in the Δ*wbaP* mutant (Fig. 4B). The Δ*wzx*_EPS_ mutant accumulated lower levels of LPS O antigen (Fig. 4B). O antigen in M. xanthus is synthesized via an ABC transporter-dependent pathway, and the lack of the Wzm/Wzt ABC transporter blocks LPS O-antigen synthesis (37, 38), suggesting that Wzx_EPS_ is not directly involved in O-antigen synthesis. Therefore, we speculate that the reduced O-antigen level in the Δ*wzx*_EPS_ mutant could be caused by sequestration of Und-PP-linked EPS intermediates unable to be translocated across the membrane, which would reduce the available pool of Und-P for O-antigen synthesis.

Interference with PG synthesis during growth in M. xanthus causes morphological defects (55–57). Therefore, we used cell morphology as a proxy for PG synthesis to test whether lack of the EPS biosynthetic proteins interferes with PG synthesis during growth. Cell morphology and cell length of the five Δ*eps* mutants were similar to that of WT cells, supporting that PG synthesis is not affected in the Δ*eps* mutants (Fig. 4A [0 h] and C). Altogether, these observations support that the Eps proteins make up a pathway dedicated to EPS synthesis.

### MXAN_7415 has Gal-1-P transferase activity.

**FIG 5:**
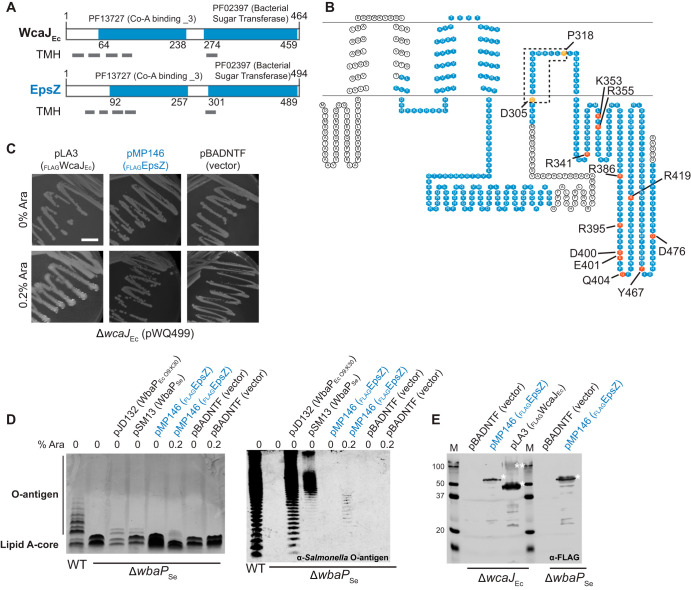
Polyisoprenyl-phosphate hexose-1-phosphate (PHPT) activity of MXAN_7415. (A) Domain and TMH prediction of EpsZ (MXAN_7415) and WcaJ of E. coli (WcaJ_Ec_). Gray rectangles indicate TMH. Numbers indicate domain borders. (B) Topology predictions for EpsZ (MXAN_7415). Domains are indicated in blue, and conserved amino acids important for structure or activity of the protein are marked with orange and red, respectively. Sequence alignment of EpsZ (MXAN_7415) with WbaP_Se_ is shown in Fig. S1. (C to E) Complementation of colanic acid synthesis and LPS O antigen in E. coli K-12 W3110 (Δ*wcaJ*_Ec_) and S. enterica LT2 (Δ*wbaP*_Se_) mutants, respectively, by plasmids encoding the indicated PHPT proteins. (C) The E. coli Δ*wcaJ*_Ec_ mutant XBF1 containing pWQ499 (RcsA^+^) and the indicated complementing plasmids or vector control on LB plates was incubated overnight at 37°C with 10 μg ml^−1^ tetracycline (to maintain pWQ499) and with or without arabinose (Ara) to induce gene expression. Incubation was extended to 24 to 48 h at room temperature to further increase colanic polysaccharide synthesis. Scale bar, 1 cm. (D) Complementation of S. enterica Typhimurium LT2 Δ*wbaP*_Se_ mutant containing the indicated plasmids. LPS samples were extracted, separated by electrophoresis on SDS–14% polyacrylamide gels, and silver stained (left) or examined by immunoblotting using rabbit *Salmonella* O antiserum group B (right). Each lane corresponds to LPS extracted from 10^8^ cells. Cultures included addition of arabinose as indicated. (E) Immunoblot using anti-FLAG monoclonal antibody to confirm expression of _FLAG_MXAN_7415 and _FLAG_WcaJ in the Δ*wcaJ* mutant and the expression of _FLAG_MXAN_7415 in S. enterica. Note that WbaP expressed from pSM13 was not tested, since it is not fused to a FLAG tag. Single and double asterisks denote the monomeric and oligomeric forms of the PHPT proteins, respectively, usually present under the gel conditions required to ensure their visualization.

EpsZ is the predicted PHPT of the EPS biosynthesis pathway. Similar to WcaJ_Ec_ from E. coli and WbaP_Se_ from Salmonella enterica (41, 58, 59), we identified a PF13727 (CoA_binding _3) domain, a C-terminal PF02397 (Bac_transf) domain, and five transmembrane regions in EpsZ (Fig. 5A), all features of PHPT proteins. The fifth TMH of WcaJ_Ec_ does not fully span the IM, and this results in the cytoplasmic localization of the C-terminal catalytic domain. This depends on residue P291, which causes a helix-break-helix in the structure and forms part of a DX_12_P motif conserved among PHPTs (59). Because EpsZ contains the DX_12_P motif and all the conserved essential residues important for catalytic activity that have been identified in the C-terminal catalytic region of WbaP (60) (Fig. 5B and Fig. S1A), we suggest that EpsZ is a PHPT with a membrane topology similar to that of WcaJ.

PHPTs generally utilize UDP-glucose (UDP-Glc) or UDP-galactose (UDP-Gal) as substrates to transfer Glc-1-P or Gal-1-P, respectively, to Und-P (29, 61). Therefore, by following the same strategy as that previously reported (37, 41, 62), we tested whether EpsZ could functionally replace WcaJ_Ec_ or WbaP_Se_, which catalyze the transfer of Glc-1-P and Gal-1-P to Und-P, respectively. To this end, *epsZ* was cloned into pBADNTF, resulting in plasmid pMP146, which encodes EpsZ with an N-terminal FLAG tag (_FLAG_EpsZ) to facilitate detection by immunoblot and under the control of an arabinose-inducible promoter.

WcaJ_Ec_ initiates colanic acid biosynthesis, which results in a strong glossy and mucoid phenotype of *wcaJ*_Ec_^+^ cells containing the plasmid pWQ499 encoding the positive regulator RcsA (59). An E. coli Δ*wcaJ*_Ec_(pWQ499) mutant is complemented with the plasmid pLA3 in the presence of arabinose (59), which encodes _FLAG_WcaJ_Ec_ under the control of the arabinose-inducible promoter (Fig. 5C and Fig. S1B). In contrast, no complementation was observed by _FLAG_EpsZ or the empty pBADNTF vector in the presence of arabinose (Fig. 5C and Fig. S1B), suggesting that EpsZ does not have Glc-1-P transferase activity.

WbaP_Se_ initiates O-antigen synthesis in S. enterica, and the O-antigen synthesis defect of a Δ*wbaP*_Se_ mutant can be partially corrected by complementation with the plasmid pJD132, which encodes the E. coli O9:K30 WbaP_Se_ homolog (WbaP_Ec O9:K30_), and with the plasmid pSM13, which encodes WbaP_Se_ (58) (Fig. 5D, left). The differences in the O-antigen profile between the different complementation strains are likely due to different processing of the O antigen, as previously reported (58). Expression of _FLAG_EpsZ in the Δ*wbaP*_Se_ mutant in the presence of arabinose provoked a change of the LPS profile (Fig. 5D, left), while the empty pBADNTF vector did not affect the LPS profile. Because the effect of _FLAG_EpsZ on the O-antigen profile of the Δ*wbaP*_Se_ mutant was relatively modest by silver staining, we repeated these experiments using *Salmonella* O-antigen rabbit antibodies. As shown in Fig. 5D, right, in this analysis, _FLAG_EpsZ complemented the Δ*wbaP*_Se_ mutant in the presence of arabinose. To test for the accumulation of _FLAG_EpsZ in the E. coli and S. enterica strains when grown in the presence of arabinose, we performed immunoblots using anti-FLAG antibodies (Fig. 5E). EpsZ accumulated in both strains predominantly in the monomeric form. In contrast, _FLAG_WcaJ_Ec_ showed the characteristic oligomeric and monomeric bands as previously reported for PHPTs (58). We conclude from these experiments that WbaP_Mx_ can transfer Gal-1-P onto Und-P.

### EPS and/or EPS biosynthetic machinery is important for T4P-dependent motility and T4P formation.

**FIG 6:**
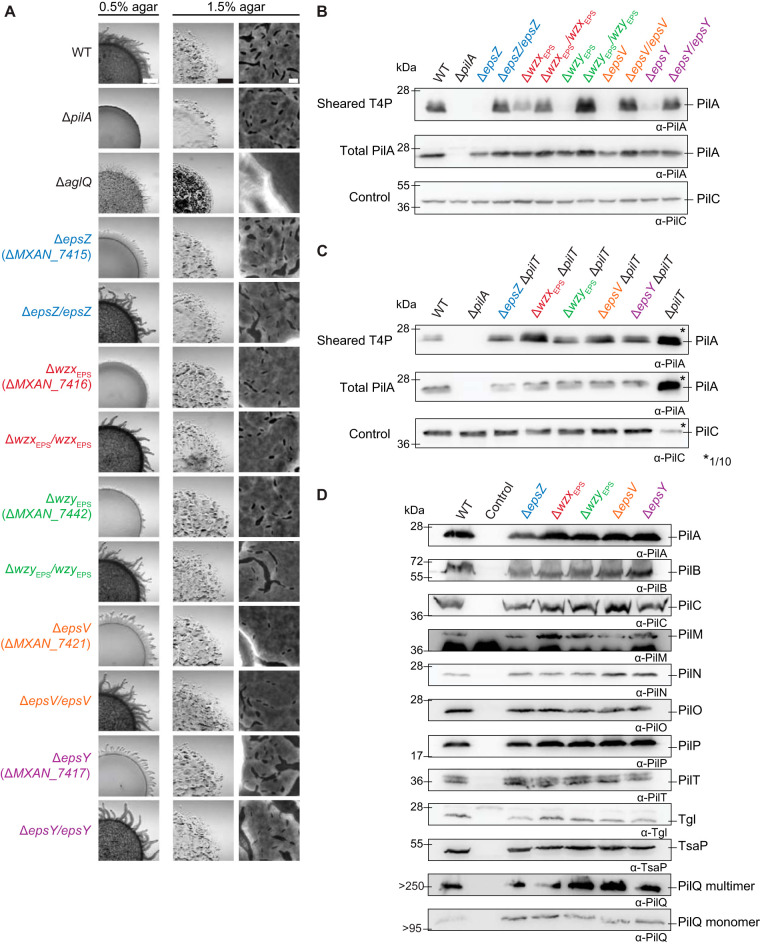
Motility analyses of Δ*eps* mutants. (A) Colony-based motility assay of Δ*eps* mutants. T4P-dependent motility and gliding motility were tested on 0.5% and 1.5% agar, respectively. Images were recorded after 24 h. Scale bars, 1 mm, 1 mm, and 10 μm (left to right). (B and C) T4P shear-off assay. Immunoblot detection of the major pilin PilA in sheared T4P (top) and in total cell extract (middle), where the same number of cells grown on 1% CTT, 1.5% agar was loaded per lane. The top and middle blots were probed with anti-PilA antibodies (calculated molecular mass, 23.4 kDa). The middle blot was stripped and probed with antibodies against PilC (calculated molecular mass, 45.2 kDa) as a loading control. (D) Immunoblot detection of proteins of the T4P machinery using anti-PilA, -B, -C, -M, -N, -O, -P, -Q, -T, -Tgl, and -TsaP antibodies. The same number of cells coming from exponentially growing liquid cultures was loaded per lane. As a negative control, cells containing a single in-frame deletion mutation in the relevant gene were used.

Next, we tested the five Δ*eps* mutants for motility defects. To this end, cells were spotted on 0.5% and 1.5% agar, respectively (14). On 0.5% agar, WT cells formed the long flares characteristic of T4P-dependent motility, while on 1.5% agar, WT displayed the single cells at the colony edge characteristic of gliding motility. The Δ*pilA* mutant, which lacks the major pilin subunit and does not assemble T4P (63), and the Δ*aglQ* mutant, which lacks a component of the gliding motility machinery (64, 65), were used as negative controls for T4P-dependent and gliding motility, respectively. As expected, the Δ*eps* mutants had a T4P-dependent motility defect, forming colonies with shorter flares than the WT, as did the Δ*aglQ* mutant (Fig. 6A). The motility defects of the Δ*eps* mutants were complemented by ectopic expression of the relevant genes (Fig. 6A). On 1.5% agar, the Δ*eps* mutants displayed the single cells at the colony edge, characteristic of gliding motility, while the Δ*aglQ* mutant did not and had a flat colony edge (Fig. 6A). The total colony expansion also was reduced similarly to that of the Δ*pilA* mutant. The reduced colony expansion of the Δ*eps* mutants was corrected in the five complementation strains (Fig. 6A). Because the Δ*aglQ* mutant made shorter flares on 0.5% agar and had no single-cell motility on 1.5% agar, the Δ*pilA* mutant made no flares on 0.5% agar and had reduced colony expansion on 1.5% agar, while the five Δ*eps* mutants generated shorter flares on 0.5% agar and still had single-cell motility on 1.5% agar, we conclude that the lack of any single one of the five EPS biosynthetic proteins causes a defect in T4P-dependent motility but not in gliding motility. Interestingly, lack of Wzx_EPS_ and Wzy_EPS_ caused a stronger defect in T4P-dependent motility than lack of EpsZ, EpsV, and EpsY (Fig. 6A).

To understand the mechanism underlying the defect in T4P-dependent motility in the Δ*eps* mutants, we determined the level of T4P formation using a shear-off assay, in which T4P are sheared off the cell surface and then the level of PilA assessed by immunoblotting. The PilA level in the sheared fraction was strongly reduced in all five Δ*eps* mutants, while the total cellular level of PilA was generally similar to that in the WT, suggesting that these mutants have fewer T4P than WT cells (Fig. 6B). Of note, the reduction in T4P-dependent motility in the five Δ*eps* mutants did not correlate with the level of T4P formation (Fig. 6A and B). Because a reduced level of T4P can result from an extension defect or hyperretraction, we deleted the *pilT* gene encoding the PilT retraction ATPase (66) in the five Δ*eps* mutants and then repeated the shearing assay. All five strains with the additional Δ*pilT* mutation assembled T4P at a higher level than the *pilT*^+^ strains but at a significantly lower level than the Δ*pilT* strain (Fig. 6C). Thus, the five Δ*eps* mutants have a defect in T4P extension. Of note, the observation that the Δ*eps pilT*^+^ strains make fewer T4P than the Δ*eps* Δ*pilT* strains support that T4P still retract in the absence of the EPS biosynthetic machinery and/or EPS.

These observations are in stark contrast to the observations for the Δ*difA* mutant, which lacks the methyl-accepting chemotaxis protein (MCP) component of the Dif system and is strongly reduced in EPS synthesis (21). This mutant was reported to make T4P at WT levels (21) or to be hyperpiliated (15), and EPS was reported to stimulate T4P retractions in this mutant (15, 19). We conclude that the lack of an EPS biosynthetic protein and/or EPS causes a reduction in T4P extension, but the fewer T4P made can still retract.

To analyze whether the reduced T4P formation in the Δ*eps* mutants was caused by reduced synthesis of one or more of the 10 core proteins of the T4P machine (13, 67) or the Tgl pilotin for PilQ (68), we determined their accumulation levels in the five *eps* mutants. All 11 proteins were detected at WT levels in the Δ*eps* mutants (Fig. 6D), suggesting that the T4P machinery is still assembled. We conclude that the EPS biosynthetic machinery and/or EPS is important for T4P extension and, therefore, T4P-dependent motility.

**FIG 7:**
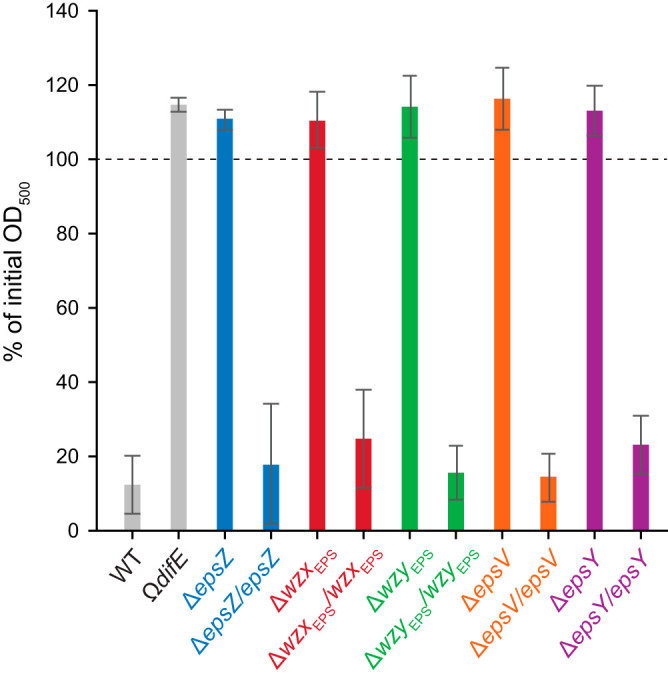
Analysis of Δ*eps* mutants for cell-cell cohesion and agglutination. Cell agglutination assay is shown. One milliliter of exponentially growing cells was transferred to a cuvette. Agglutination was monitored by measuring the decrease in absorbance at 550 nm at 3 h relative to the initial absorbance for each strain. The graph shows data from three biological replicates as means ± standard deviations.

Cell-cell cohesion has been suggested to depend on EPS (10, 16, 69). To evaluate whether the Δ*eps* mutants were affected in cell-cell cohesion and agglutination, we transferred exponentially growing cells to a cuvette and measured the change in cell density over time. WT cells agglutinated and sedimented during the course of the experiment, causing a decrease in the absorbance (Fig. 7). Ω*difE* and a mutant were used as a negative control and did not agglutinate over time (21). None of the five Δ*eps* in-frame deletion strains agglutinated (Fig. 7), and the agglutination defect was complemented in the complementation strains (Fig. 7).

### EPS and/or the EPS biosynthetic machinery is conditionally important for fruiting body formation.

**FIG 8:**
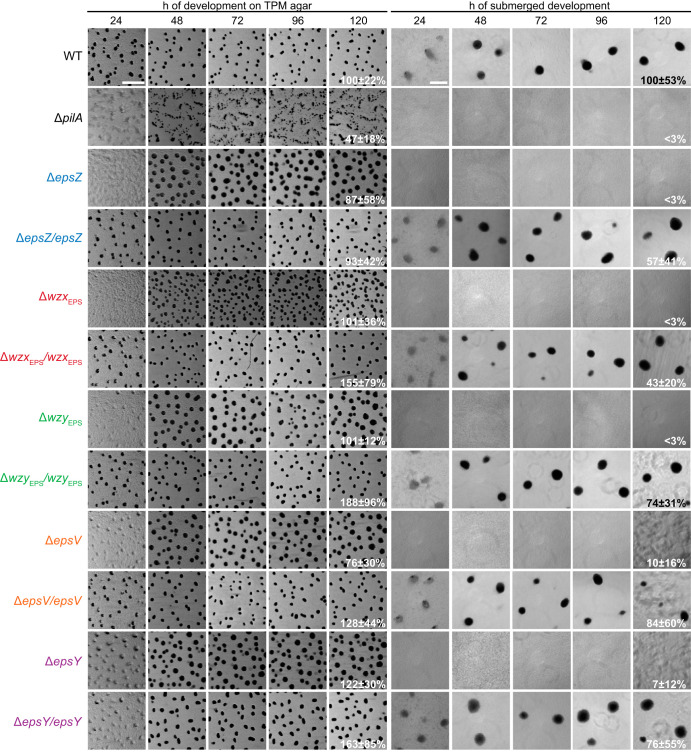
Development of Δ*eps* mutants. Cells on TPM agar and under submerged conditions were monitored during development. Images were recorded at the indicated time points. Sporulation efficiency after heat and sonic treatment is indicated as the means ± standard deviations from three biological replicates relative to the WT. Scale bars, 1 mm (left) and 200 μm (right).

Next, we tested the five Δ*eps* mutants for development. On TPM agar (10 mM Tris-HCl [pH 7.6], 1 mM K_2_HPO_4_-KH_2_PO_4_ [pH 7.6], 8 mM MgSO_4_) and in submerged culture, WT cells had aggregated to form darkened mounds at 24 h of starvation (Fig. 8). On TPM agar, the Δ*eps* mutants showed a delay in aggregation but eventually formed larger and less compact fruiting bodies and sporulated with an efficiency similar to that of the WT (Fig. 8). Under submerged conditions, the Δ*eps* mutants did not aggregate to form fruiting body sporulation, as expected from the cell-cell cohesion and agglutination defects, and were significantly reduced in sporulation. The developmental defects of the five Δ*eps* mutants were largely restored by ectopic expression of the corresponding gene (Fig. 8). These observations are also in stark contrast to the observations for *dif* mutants with an EPS̄ phenotype, which do not aggregate on solid surfaces (17, 18).

## DISCUSSION

Here, we focused on elucidating key steps of EPS biosynthesis and determined functional consequences of the loss of the EPS biosynthetic machinery. The EPS structure is unknown; however, chemical analyses support that it contains at least *N*-acetylglucosamine (GlcNAc), Glc, and Gal, while data for other monosaccharides vary depending on the analysis (70, 71).

**FIG 9:**
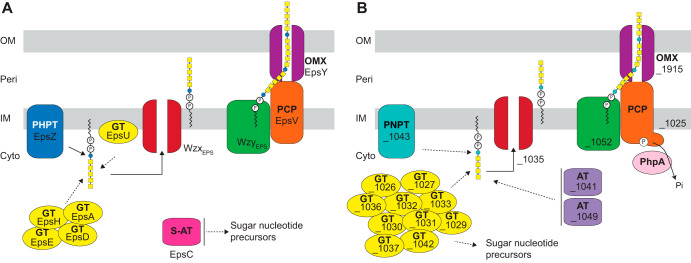
Wzx/Wzy-dependent pathways for EPS biosynthesis (A) and synthesis of an unknown polysaccharide (B). See Discussion for details. Cyto, cytoplasm; Peri, periplasm.

Using bioinformatics, we identified the genes for all the components of a Wzx/Wzy pathway in the *eps* locus. Our experimental results support a model in which these genes encode the EPS biosynthesis machinery (Fig. 9A) and that synthesis of the EPS repeat unit is initiated by the PHPT homolog EpsZ (MXAN_7415). We demonstrate in heterologous expression experiments that EpsZ is functionally similar to the Gal-1-P transferase WbaP_Se_, suggesting that Gal is the first sugar of the EPS repeat unit. The *eps* locus encodes five GTs, and inactivation of each of these five genes (9, 34, 42) causes a loss of EPS synthesis or T4P-dependent motility (Fig. 2A). Therefore, we suggest that these five GTs add monosaccharides to build the repeat unit, which is then translocated across the IM by the Wzx_EPS_ flippase (MXAN_7416). The repeat units are polymerized by the Wzy_EPS_ polymerase (MXAN_7442) with the help of the PCP protein EpsV (MXAN_7421) to make the EPS polysaccharide. In the last step, the EPS polymer is transported to the surface through the OPX protein EpsY (MXAN_7417). EpsC (MXAN_7449) is a serine *O*-acetyltransferase homolog, which is important but not essential for EPS synthesis (9). As previously suggested for a paralog encoded by *exoN* (41), which is important for spore coat polysaccharide synthesis, MXAN_7449 could be involved in O-acetylation of precursors for EPS synthesis. Finally, the predicted glycosyl hydrolase EpsB (MXAN_7450) is also important but not essential for EPS synthesis (9), and its biochemical function remains to be characterized. Overall, our genetic and functional analyses support that the EPS biosynthesis machinery is exclusively dedicated to EPS biosynthesis and not involved in LPS O-antigen or spore coat polysaccharide biosynthesis.

We also identified two additional loci, which together encode a complete Wzx/Wzy-dependent pathway (Fig. 9B). Our genetic analysis suggests the proteins of this pathway are not involved in EPS biosynthesis and spore coat polysaccharide and LPS O-antigen synthesis (Fig. 3D and unpublished data), indicating a novel function. While the manuscript was in preparation, Islam et al. (72) reported that this pathway synthesizes a biosurfactant that is important for T4P-dependent motility.

Genetic analyses of the five core components of the EPS biosynthesis machinery showed that the lack of any of these proteins caused a defect not only in EPS synthesis but also in T4P-dependent motility and cell-cell cohesion, as well as a conditional defect in fruiting body formation. Superficially, these defects are similar to those reported for *dif* mutants with an EPS̄ phenotype, which are the best-studied mutants with decreased EPS synthesis. However, more detailed comparisons reveal important differences. First, *dif* mutants with an EPS̄ phenotype have a defect in T4P-dependent motility (17, 18); however, a *difA* mutant makes T4P at WT levels (21) or is hyperpiliated (15). Moreover, it was suggested that EPS stimulates T4P retractions in this mutant, because the addition of EPS caused reduced piliation (15, 19). Because the *dif* mutants with an EPS̄ phenotype make T4P but have reduced T4P-dependent motility, this supports that EPS *per se* might stimulate T4P-dependent motility. In contrast, we observed that the five Δ*eps* mutants analyzed here are hypopilated. Further deletion of the gene for the PilT retraction ATPase also resulted in an increased level of surface piliation, suggesting that T4P in the five Δ*eps pilT^+^* mutants can still be retracted. Consistent with this, T4P-dependent motility was not completely abolished in the five Δ*eps* mutants. These observations suggest that EPS, or, alternatively, components of the EPS biosynthetic machinery, is important for T4P formation. Altogether, these comparisons support that the *dif* EPS̄ mutations, which are regulatory mutants, and the Δ*eps* mutations described here, which are biosynthetic mutants, both interfere with T4P-dependent motility, but the underlying mechanisms are different. Second, *dif* mutants with an EPS̄ phenotype develop to form spore-filled fruiting bodies neither on TPM or CF agar nor under submerged conditions (17, 18, 43). Of note, development of such mutants on TPM agar was rescued by addition of EPS (21, 73). In contrast, the five Δ*eps* mutants described here develop with only a slight delay on TPM agar but not under submerged conditions. We speculate that this developmental defect is caused by lack of cell-cell cohesion and agglutination in the five Δ*eps* mutants. Whether these phenotypic differences are caused by the differences in T4P levels and functionality in the two types of mutants remains to be investigated.

Previously, it was reported that the T4P machinery functions upstream of the Dif pathway to stimulate EPS synthesis (74–77). How the T4P machinery interfaces with the Dif system is unknown. Similarly, it is unknown how the Dif system stimulates EPS biosynthesis. Here, we show that mutations in the Wzx/Wzy-dependent pathway for EPS synthesis cause a defect in T4P extension. How this effect is brought about remains to be determined. Interestingly, different Δ*eps* mutations had different effects on T4P extension, indicating that the extension defect is not simply caused by lack of EPS. Because all five Δ*eps* mutants accumulate all the proteins of the T4P machine and this machine is at least partially functional, we speculate that the EPS machinery, possibly together with EPS, stimulate the function of the T4P machine. It will be an important future goal to disentangle how *dif* and *eps* mutants at the molecular level affect T4P formation and function as well as how the T4P machinery affects EPS synthesis.

## MATERIALS AND METHODS

### Strains and cell growth.

All M. xanthus strains are derivatives of the wild-type DK1622 (78). Strains, plasmids, and oligonucleotides used in this work are listed in Tables 1 and 2 and Table S3 in the supplemental material, respectively. M. xanthus was grown at 32°C in 1% CTT (1% [wt/vol] Bacto Casitone, 10 mM Tris-HCl [pH 8.0], 1 mM K_2_HPO_4_-KH_2_PO_4_ [pH 7.6], and 8 mM MgSO_4_) liquid medium or on 1.5% agar supplemented with 1% CTT and kanamycin (50 μg ml^−1^) or oxytetracycline (10 μg ml^−1^), as appropriate (79). In-frame deletions were generated as described previously (80), and plasmids for complementation experiments were integrated in a single copy by site-specific recombination into the Mx8 *attB* site. In-frame deletions and plasmid integrations were verified by PCR. Plasmids were propagated in E. coli Mach1 and DH5α.

**TABLE 1:** Strains used in this work

Strain	Genotype	Reference(s) or source
M. xanthus		
DK1622	WT	78
DK8615	Δ*pilQ*	105
DK10405	Δ*tg*l	106, 107
DK10409	Δ*pilT*	66, 90
DK10410	Δ*pilA*	90
DK10416	Δ*pilB*	66, 90
DK10417	Δ*pilC*	90
SW501	*difE*::Km^r^	17
SA3001	Δ*pilO*	87
SA3002	Δ*pilM*	86
SA3005	Δ*pilP*	87
SA3044	Δ*pilN*	87
SA5923	Δ*aglQ*	108
SA6011	Δ*tsaP*	88
SA7450	Δ*wbaP*_Mx_	37
SA7495	Δ*exoE*	37
SA7400	Δ*MXAN_7415*	This study
SA7405	Δ*MXAN_7416*	This study
SA7406	Δ*MXAN_7421*	This study
SA7407	Δ*MXAN_7442*	This study
SA7408	Δ*MXAN_7417*	This study
SA7410	Δ*MXAN_7416 attB*::pMP024 (P_nat_ *MXAN_7416*)	This study
SA7411	Δ*MXAN_7415 attB*::pMP021 (P_nat_ *MXAN_7415*)	This study
SA7412	Δ*MXAN_7417 attB*::pMP030 (P_pilA_ *MXAN_7417*)	This study
SA7413	Δ*MXAN_7421 attB*::pMP032 (P_pilA_ *MXAN_7421*)	This study
SA7427	Δ*MXAN_7416 ΔpilT*	This study
SA7433	Δ*MXAN_7415 ΔpilT*	This study
SA7435	Δ*MXAN_7442 ΔpilT*	This study
SA7444	Δ*MXAN_7417 ΔpilT*	This study
SA7445	Δ*MXAN_7421 ΔpilT*	This study
SA7451	Δ*MXAN_1025*	This study
SA7452	Δ*MXAN_1035*	This study
SA7456	Δ*MXAN_1052*	This study
SA7454	Δ*MXAN_1915*	This study
SA7477	Δ*MXAN_7442 attB*::pMP091 (P_nat_ *MXAN_7442*)	This study
SA8515	Δ*MXAN_1043*	This study
E. coli		
DH5α	F^−^ ϕ80*lacZ*ΔM15 *endA recA hsdR*(r_K_^−^ m_K_^−^) *nupG thi glnV deoR gyrA relA1* Δ(*lacZYA-argF*)*U169*	Laboratory stock
Mach1	Δ*recA1398 endA1 tonA* ϕ80Δ*lac*M15 Δ*lacX74 hsdR*(r_K_^−^ m_K_^+^)	Invitrogen
XBF1	W3110 *ΔwcaJ*::*aph* Km^r^	62
*Salmonella*		
LT2	WT S. enterica serovar Typhimurium	S. Maloy
MSS2	LT2 Δ*wbaP*::*cat* Cm^r^	58

**TABLE 2:** Plasmids used in this work

Plasmid	Description	Reference or source
pBJ114	Km^r^ *galK*	109
pSWU30	Tet^r^	63
pSW105	Km^r^ PpilA	66
pBADNTF	pBAD24 for N-terminal FLAG fusion and with arabinose-inducible promoter, Amp^r^	110
pLA3	pBADNTF *wcaJ* Amp^r^	59
pSM13	pUC18, *wbaP* from S. enterica Ty2 containing a 1-bp deletion at position 583 a 2-bp deletion at position 645, which causes a frame shift at WbaP I194 and frame restoration at Y215, Amp^r^	58
pJD132	pBluescript SK, *wbaP* and flanking sequences from E. coli O9 : K30, Amp^r^	111
pWQ499	pKV102 containing *rcsAK30*, Tet^r^	Chris Whitfield
pMAT150	pBJ114, in-frame deletion construct for *pilT* Km^r^	Anke Treuner-Lange
pMP001	pBJ114, in-frame deletion construct for *MXAN_7415* Km^r^	This study
pMP012	pBJ114, in-frame deletion construct for *MXAN_7421* Km^r^	This study
pMP015	pBJ114, in-frame deletion construct for *MXAN_7442* Km^r^	This study
pMP016	pBJ114, in-frame deletion construct for *MXAN_7416* Km^r^	This study
pMP018	pBJ114, in-frame deletion construct for *MXAN_7417* Km^r^	This study
pMP021	pSWU30 P_nat_ *MXAN_7415* Tet^r^	This study
pMP024	pSWU30 P_nat_ *MXAN_7416* Tet^r^	This study
pMP030	pSW105 *MXAN_7417* Km^r^	This study
pMP032	pSW105 *MXAN_7421* Km^r^	This study
pMP091	pSWU30 P_nat_ *MXAN_7442* Tet^r^	This study
pMP124	pBJ114, in-frame deletion construct for *MXAN_1043* Km^r^	This study
pMP146	pBADNTF MXAN_7415 Amp^r^	This study
pJJ1	pBJ114, in-frame deletion construct for *MXAN_1035* Km^r^	This study
pJJ2	pBJ114, in-frame deletion construct for *MXAN_1025* Km^r^	This study
pJJ3	pBJ114, in-frame deletion construct for *MXAN_1052* Km^r^	This study
pJJ4	pBJ114, in-frame deletion construct for *MXAN_1915* Km^r^	This study

E. coli and S. enterica serovar Typhimurium strains were grown at 37°C in Luria-Bertani (LB) medium (10 mg tryptone ml^−1^, 5 mg yeast extract ml^−1^, 5 mg NaCl ml^−1^) supplemented, when required, with ampicillin, tetracycline, kanamycin, or chloramphenicol at a final concentration of 100, 20, 40, or 30 μg ml^−1^, respectively. Plasmids for heterologous complementation were introduced into MSS2 and XBF1 strains (Table 1) by electroporation (81).

### Detection of EPS accumulation.

Exponentially growing cells were harvested (3 min, 6,000 × *g* at room temperature [RT]) and resuspended in 1% CTT to a calculated density of 7 × 10^9^ cells ml^−1^. Twenty-microliter aliquots of the cell suspensions were placed on 0.5% agar plates supplemented with 0.5% CTT and 10 or 20 μg ml^−1^ of trypan blue or Congo red, respectively. Plates were incubated at 32°C and documented at 24 h.

### Glycerol-induced sporulation assay.

Sporulation in response to 0.5 M glycerol was performed as described previously (82), with a slightly modified protocol. Briefly, cells were cultivated in 10 ml of CTT medium at a cell density of 3 × 10^8^ cells ml^−1^, and glycerol was added to a final concentration of 0.5 M. At 0, 4, and 24 h after glycerol addition, cell morphology was observed by placing 5 μl of cells on a 1.5% agar TPM pad on a slide. Cells were immediately covered with a coverslip and imaged with a DMi6000B microscope and a Hamamatsu Flash 4.0 camera (Leica). To determine the resistance to heat and sonication of spores formed, cells from 5 ml of the culture after 24 h of incubation were harvested (10 min, 4,150 × *g*, RT), resuspended in 1 ml sterile water, incubated at 50°C for 2 h, and then sonicated with 30 pulses (pulse, 50%; amplitude, 75%; with a UP200St sonifier and microtip; Hielscher). Sporulation levels were determined as the number of sonication- and heat-resistant spores relative to the WT using a Helber bacterial counting chamber (Hawksley, UK), and 0.5 μl of the treated samples was placed on a 1.5% agar TPM pad on a slide, covered with a coverslip, and imaged.

### LPS extraction and detection.

LPS was extracted from M. xanthus and visualized by Emerald staining as described previously (37). LPS from S. enterica and E. coli was extracted and visualized by silver staining as described previously (37, 83). For S. enterica, O antigen was detected by immunoblot using rabbit *Salmonella* O antiserum group B (number 229481; Difco, Becton Dickinson) (1:500) and the secondary antibody IRDye 800CW goat anti-rabbit immunoglobulin G (1:10,000) (LI-COR) (37).

### Cell length determination.

Five-microliter aliquots of exponentially growing cell suspensions were spotted on glass placed on a metal frame, covered with 1.5% agar supplemented with TPM, and imaged using a DMi8 inverted microscope and DFC9000 GT camera (Leica) (84). Cell length was determined and visualized as described previously (37). Statistical analyses were performed using SigmaPlot v14. All data sets were tested for a normal distribution using a Shapiro-Wilk test, and for all data sets without a normal distribution, the Mann-Whitney test was applied to test for significant differences.

### Motility assays.

Exponentially growing cultures of M. xanthus were harvested (6,000 × *g*, RT) and resuspended in 1% CTT to a calculated density of 7 × 10^9^ cells ml^−1^. Five-microliter aliquots of cell suspensions were spotted on 0.5% and 1.5% agar supplemented with 0.5% CTT. The plates were incubated at 32°C for 24 h, and cells were visualized using a M205FA stereomicroscope (Leica) and imaged using a Hamamatsu ORCA-flash V2 digital CMOS camera (Hamamatsu Photonics). Pictures were analyzed using Metamorph v 7.5 (Molecular Devices).

### Detection of colanic acid biosynthesis.

E. coli Δ*wcaJ* strains were grown on LB plates with antibiotics and with or without 0.2% (wt/vol) arabinose at 37°C overnight. Incubation was extended to 24 to 48 h at RT to visualize the mucoid phenotype (Furlong et al. [59]).

### Immunoblot analysis.

Immunoblots were carried out as described previously (85). For M. xanthus immunoblots, rabbit polyclonal anti-PilA (dilution, 1:2,000), anti-PilB (dilution, 1:2,000) (66), anti-PilC (dilution, 1,2,000) (86), anti-PilM (dilution, 1:3,000) (86), anti-PilN (dilution, 1:2,000) (87), anti-PilO (dilution, 1:2,000) (87), anti-PilP (dilution, 1:2,000) (87), anti-PilT (dilution, 1:3,000) (66), anti-Tgl (dilution, 1:2,000) (87), anti-TsaP (dilution, 1:2,000) (88), and anti-PilQ (dilution, 1:5,000) (86) were used together with a horseradish-conjugated goat anti-rabbit immunoglobulin G (Sigma) as a secondary antibody. Blots were developed using Luminata crescendo Western HRP substrate (Millipore) on a LAS-4000 imager (Fujifilm).

For E. coli and S. enterica strains, FLAG-tagged membrane proteins were isolated and detected by immunoblot analysis, as previously described, using anti-FLAG M2 monoclonal antibody (Sigma) (1:10,000) and a secondary antibody, 0.5 mg IRDye 800CW goat anti-mouse IgG (H+L) (1:10,000) (LI-COR) (37).

### T4P shear-off assay.

T4P were sheared from cells that had been grown for 3 days on 1.5% agar plates supplemented with 1% CTT at 32°C as described above, except that precipitation of sheared T4P was done using trichloroacetic acid as described previously (89) and analyzed by immunoblotting with anti-PilA antibodies as described previously (63). Blots were developed as indicated.

### Cell agglutination assay.

Cell agglutination was performed as described previously (90), with a slightly modified protocol. Briefly, 1 ml of exponentially growing cells in 1% CTT was transferred to a cuvette, and cell density was measured at the indicated time points.

### Development.

Exponentially growing M. xanthus cultures were harvested (3 min, 6,000 × *g* at RT) and resuspended in MC7 buffer (10 mM morpholinepropanesulfonic acid [pH 7.0], 1 mM CaCl_2_) to a calculated density of 7 × 10^9^ cells ml^−1^. Ten-microliter aliquots of cells were placed on TPM agar (10 mM Tris-HCl [pH 7.6], 1 mM K_2_HPO_4_-KH_2_PO_4_ [pH 7.6], 8 mM MgSO_4_), and 50-μl aliquots were mixed with 350 μl of MC7 buffer and placed in a 24-well polystyrene plate (Falcon) for development in submerged culture. Cells were visualized at the indicated time points using an M205FA stereomicroscope (Leica) and imaged using a Hamamatsu ORCA-flash V2 digital CMOS camera (Hamamatsu Photonics), DMi8 inverted microscope, and DFC9000 GT camera (Leica). Images were analyzed as previously described. After 120 h, cells were collected and incubated at 50°C for 2 h and then sonicated as described above. Sporulation levels were determined as the number of sonication- and heat-resistant spores relative to the WT.

### Bioinformatics.

The KEGG SSDB (Sequence Similarity Database) (91) database was used to identify homologs of PHPT (PF02397, Bacterial Sugar Transferase), PNPT (PF00953, Glycosyl transferase family 4) (92), Wzx (PF01943, Polysacc_synt, and PF13440, Polysacc_synt_3), Wzy_C (PF04932, Wzy_C), PCP (PF02706, Wzz), and OPX (PF02563, Poly_export), as described previously (41, 93, 94). For the ABC transporter-dependent pathway we used (PF01061, ABC2_membrane) for the permease and, (PF00005, ABC_tran) and (PF14524, Wzt_C) for the ATPase, as described in reference 37, together with an analysis of the genetic neighborhood to search for glycan-related proteins. BLASTP was used to identify homologs of the synthase-dependent pathway using previously identified components (33). KEGG SSDB was also used to identify EPS homolog proteins in other *Myxococcales* using a reciprocal best BLASTP hit method. UniProt (95), KEGG (91), and the Carbohydrate Active Enzymes (CAZy) (http://www.cazy.org/) (96) databases were used to assign functions to proteins (Fig. 1B, 2A, 3A and B, and Tables S1 and S2). SMART (smart.embl-heidelberg.de) (97) and Pfam v31.0 and v32.0 (pfam.xfam.org) (98) were used to identify protein domains. Membrane topology was assessed by TMHMM v2.0 (99), and two-dimensional topology was graphically shown using TOPO2 (100). Clustal Omega (101) was used to align protein sequences. The phylogenetic tree was prepared as described in reference 41 in MEGA7 (102) using the neighbor-joining method (103). Bootstrap values (500 replicates) are shown next to the branches (104).

### Data availability.

The data that support the findings of this study are available from the corresponding author upon request.

## Supplementary Material

Supplemental file 1
